# Twitter Sentiment Predicts Affordable Care Act Marketplace Enrollment

**DOI:** 10.2196/jmir.3812

**Published:** 2015-02-23

**Authors:** Charlene A Wong, Maarten Sap, Andrew Schwartz, Robert Town, Tom Baker, Lyle Ungar, Raina M Merchant

**Affiliations:** ^1^Robert Wood Johnson Foundation Clinical Scholars ProgramUniversity of PennsylvaniaPhiladelphia, PAUnited States; ^2^Penn Medicine Social Media and Health Innovation LabUniversity of PennsylvaniaPhiladelphia, PAUnited States; ^3^Leonard Davis Institute of Health EconomicsUniversity of PennsylvaniaPhiladelphia, PAUnited States; ^4^Computer & Information Science Department and Positive Psychology CenterUniversity of PennsylvaniaPhiladelphia, PAUnited States; ^5^The Wharton SchoolUniversity of PennsylvaniaPhiladelphia, PAUnited States; ^6^National Bureau of Economic ResearchCambridge, MAUnited States; ^7^Penn Law SchoolUniversity of PennsylvaniaPhiladelphia, PAUnited States; ^8^Department of Emergency MedicineUniversity of PennsylvaniaPhiladelphia, PAUnited States

**Keywords:** affordable care act, social media, health insurance marketplace

## Abstract

**Background:**

Traditional metrics of the impact of the Affordable Care Act (ACA) and health insurance marketplaces in the United States include public opinion polls and marketplace enrollment, which are published with a lag of weeks to months. In this rapidly changing environment, a real-time barometer of public opinion with a mechanism to identify emerging issues would be valuable.

**Objective:**

We sought to evaluate Twitter’s role as a real-time barometer of public sentiment on the ACA and to determine if Twitter sentiment (the positivity or negativity of tweets) could be predictive of state-level marketplace enrollment.

**Methods:**

We retrospectively collected 977,303 ACA-related tweets in March 2014 and then tested a correlation of Twitter sentiment with marketplace enrollment by state.

**Results:**

A 0.10 increase in the sentiment score was associated with an 8.7% increase in enrollment at the state level (95% CI 1.32-16.13; *P*=.02), a correlation that remained significant when adjusting for state Medicaid expansion (*P*=.02) or use of a state-based marketplace (*P*=.03).

**Conclusions:**

This correlation indicates Twitter’s potential as a real-time monitoring strategy for future marketplace enrollment periods; marketplaces could systematically track Twitter sentiment to more rapidly identify enrollment changes and potentially emerging issues. As a repository of free and accessible consumer-generated opinions, this study reveals a novel role for Twitter in the health policy landscape.

## Introduction

The Patient Protection and Affordable Care Act (ACA), often referred to as Obamacare, established health insurance marketplaces in 2013 to extend coverage to more Americans. The health insurance marketplaces, also known as “exchanges”, are Web-based platforms where consumers can compare and purchase health insurance plans [1]. In the marketplaces’ first year, the majority of US states used the federal marketplace on HealthCare.gov (eg, federally facilitated marketplace), while 17 states set up their own health insurance exchange websites (eg, state-based marketplace) [2]. However, the ACA has been publicly debated, and the marketplaces struggled with technical issues in the first open enrollment period from October 2013 to April 2014, which was the time period designated for consumers to purchase a new health insurance plan in the marketplaces [3-10].

With such monumental and sometimes controversial changes, both supporters and opponents awaited measures of success or failure that included surveys of public support for the ACA and reports of marketplace enrollment, meaning the number of people purchasing health insurance plans in the marketplace (eg, marketplace plans) by state [11-14]. The release of these traditional metrics, however, lagged weeks to months. This delay was particularly relevant for the marketplaces as federal and state agencies worked to understand what was happening and address issues as they arose. In this rapidly changing environment, a real-time barometer of public opinion with a mechanism to identify emerging issues would have been valuable.

Twitter, an online micro-blogging social media outlet that allows for measure of public sentiment, is a potential new tool to monitor the rollout of major health policy. Twitter sentiment (the positivity or negativity of tweets) has previously been used to measure public perception on a range of health topics, from disease outbreaks and disaster response to health care quality and health reform [15-18]. In the debate around the ACA, Twitter became a prominent platform for scrutiny and praise. Twitter members used hashtag terms #ACA and #Obamacare to track these conversations. In this study, we sought to evaluate Twitter’s role as a real-time barometer of public sentiment on the ACA and to determine if Twitter sentiment could be predictive of state-level marketplace enrollment.

## Methods

### Overview

To evaluate the relationship between Twitter sentiment and marketplace enrollment, we retrospectively collected ACA-related tweets by state and then tested a correlation of Twitter sentiment with marketplace enrollment by state.

### Twitter Data Collection

We collected ACA-related tweets from March 1-31, 2014, using the Twitter Search Application Programming Interface [19]. Specifically, we selected all tweets containing the terms “ACA”, “#ACA”, “Obamacare”, and “#Obamacare” (a total of 977,303 tweets) as well as those that were directed toward the Twitter account handles for HealthCare.gov (eg, @HealthCaregov) and the 17 state-based marketplace Twitter accounts (an additional 34,605 tweets; see Multimedia Appendix 1). Additionally, we collected a random sample of 977,303 tweets from March 2014 to use as a comparison group for the sentiment of ACA and Obamacare tweets.

Tweet content and geolocation data, when available, were extracted. Twitter provides latitude and longitude coordinates or the self-reported location of the Twitter user. For the self-reported location, we matched the location text with state names, state abbreviations, and the 60 most populated cities in the United States. To verify this automatic technique, we manually examined 400 randomly selected mappings and found 99% accuracy.

### Twitter Sentiment

Twitter sentiment was measured using the National Research Council (NRC) sentiment lexicon [20]. The lexicon contains a list of 54,120 words along with sentiment weights ranging from positive values for positive sentiment to negative values for negative sentiment (eg, the word “excellent” has a positive sentiment weight, while “awful” is negative). The NRC lexicon was created in a data-driven fashion by analyzing tweets with positive and negative sentiment hashtags. Specifically, Mohammad et al use the point-wise mutual information metric to find an association between words and their being a part of a tweet with a positive or negative sentiment hashtag [20]. The lexicon was validated against a hand-annotated set of tweets as part of the SemEval-2012 sentiment task and was found to perform with an F1 value of 0.65. We are not aware of any lexicon achieving better accuracy over a standard set of Twitter data.

To apply the lexicon to a tweet, we computed the *relative frequency* for each word in the tweet (eg, the word frequency divided by total number of words in the tweet) and *word sentiment scores* per tweet by multiplying the *sentiment weight* by the *relative frequency* of each word. A single sentiment score for each tweet was produced by summing all *word sentiment scores* for the tweet. This is illustrated in the following equation where *sentiment(word)* is the NRC lexicon sentiment score for the word, *frequency *is the number of times the word occurred in the tweet, and *frequency_all_words* gives the number of (non unique) word instances in the tweet:

*sentiment score(tweet) =* Σ _wordεtweet_ sentiment(word) * frequency(word, tweet)/frequency_all_words(tweet)

These scores were then standardized by *Z* scores, and a state’s sentiment score was calculated as the average sentiment of tweets in the state.

The NRC sentiment lexicon has been used to produce state-of-the-art accuracies for general domain tweets [20]. However, because our ACA corpus of tweets is biased compared to general tweets, we validate the NRC sentiment lexicon over our tweets. We randomly sampled 300 tweets from our corpus and had 2 raters score the sentiment from -3 (extremely negative) to 0 (neutral) to 3 (extremely positive); interrater reliability was strong (intraclass correlation=.72). We found the NRC sentiment lexicon was significantly correlated with the mean of the human ratings (*r*=.26; *P*<.001).

### Marketplace Enrollment Data

We used health insurance marketplace enrollment data by state from the Office of the Assistant Secretary for Planning and Evaluation and the Kaiser Family Foundation, which provides the number of people who selected a new health insurance plan in the marketplaces through March 31 and the special extended enrollment period through April 19, 2014 [12,21]. The total number of consumers eligible to enroll in a marketplace plan included legally residing individuals who were uninsured or purchased non-group coverage, had incomes above Medicaid/CHIP eligibility levels, and who did not have access to employer-sponsored coverage. The estimate excluded uninsured individuals with incomes below the federal poverty level in states that did not expand Medicaid [12,22]. The percent eligible who selected a plan represented the number of participants who selected a marketplace plan (with or without receipt of the first premium payment) divided by the total number of consumers eligible to enroll in a marketplace plan.

### Data Analysis

Descriptive statistics and paired *t* tests were used to assess Twitter sentiment for ACA and Obamacare tweets, tweets directed to (eg @) the HealthCare.gov handle and state-based marketplace handles, and the random sample of comparator tweets. State Twitter sentiment for tweets containing ACA, #ACA, Obamacare, or #Obamacare were mapped using ArcGIS version 10.1.

The correlation between sentiment for ACA and Obamacare tweets and the percent of eligible individuals who selected a marketplace plan was assessed using a linear regression with robust standard errors. Adjusted analysis controlling for state Medicaid expansion [22] and if a state had a state-based or federally facilitated marketplace [2] were also conducted. Vermont was excluded as an outlier since their enrollment rate was over 2.5 standard deviations above the mean.

All analyses were conducted using STATA 13.1. The study was deemed exempt by the University of Pennsylvania Institutional Review Board.

## Results

Sentiment for ACA and Obamacare tweets was significantly more negative compared to the random sample of tweets (0.44 standard deviations [SD] lower, *P*<.001). Tweets containing ACA or #ACA had a higher sentiment compared to those with Obamacare or #Obamacare (0.46 SD higher; *P*<.001). The sentiment of tweets at the HealthCare.gov handle was 0.20 SD lower than tweets at the state-based marketplace handles (*P*<.001). Table 1 provides examples of positive and negative sentiment tweets.

**Figure 1:**
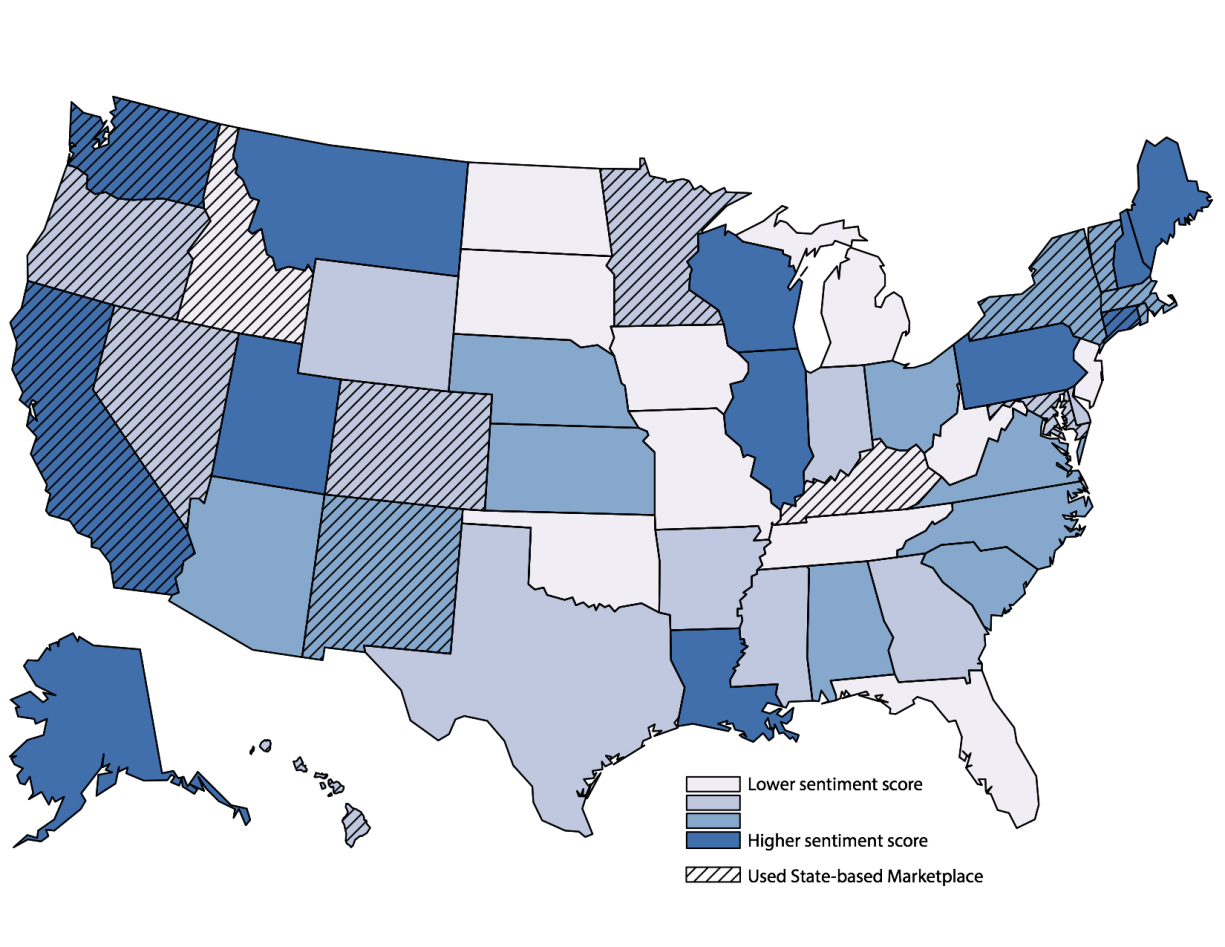
Sentiment of tweets about the Affordable Care Act or Obamacare by state, March 2014. Twitter sentiment (the positivity or negativity of tweets with higher sentiment scores indicating more positive sentiment) are presented in quartiles.

Of the 977,303 tweets, 449,553 (46.00%) were geocoded to the state level. Figure 1 shows the state-level distribution of Twitter sentiment for ACA and Obamacare. The mean percent eligible enrolled across states was 23.5% (SD 11.7%).

**Figure 2:**
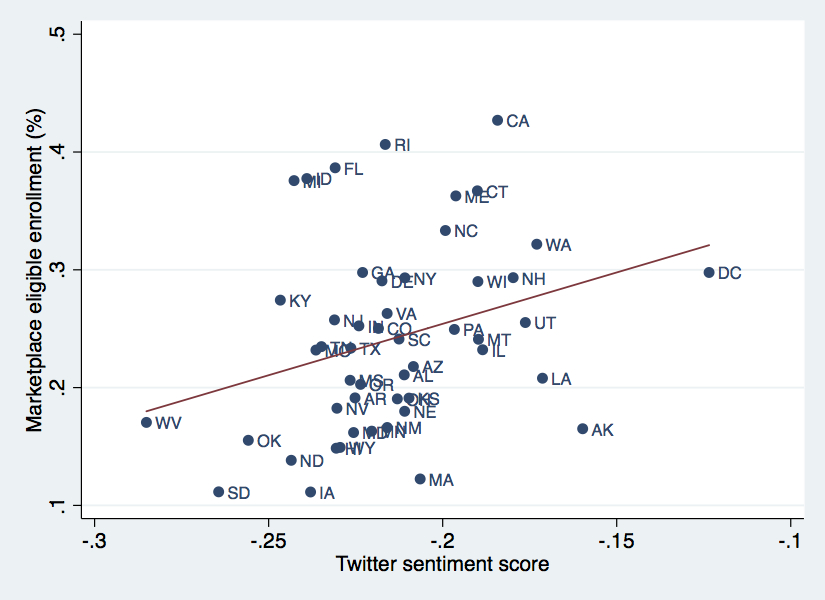
Correlation between Twitter sentiment and health insurance marketplace enrollment by state, March 2014. Abbreviations indicate US state. Vermont excluded due to outlier marketplace enrollment.

A 0.10 increase in sentiment score was associated with an 8.7% increase in enrollment at the state level (95% CI 1.32-16.13; *P*=.02) (Figure 2). The correlation remained significant when adjusting for state Medicaid expansion (*P*=.02) or whether states had a state-based or federally facilitated marketplace (*P*=.03).

**Table 1:** Sample tweets with positive and negative sentiment for ACA, Obamacare, and the health insurance marketplaces.

	Positive sentiment	Negative sentiment
#ACA or ACA	It's such a relief to have AFFORDABLE health insurance while just working part-time. My policy starts 4/1 and I'm so grateful for the ACA!	His 38% approval belies an unprecedented level of distrust. His hyper partisanship has destroyed his credibility! @GOP #IRS #ACA #Benghazi
	Another family covered under ACA in Texas -- my son & his family with pre-existing conditions of asthma & epilepsy! #ACAworks	RT If you are a Victim of ObamaCare #ACA #ObamaCare #CNN #Tcot #Pjnet #Hannity #NRA #Benghazi #NSA #IRS @CNN #Obama
Obamacare or #Obamacare	Healthy citizens are the greatest asset any country can have. ~Winston Churchill~ #GetCovered #ObamaCare	Once again @BarackObama is breaking the law by telling illegals to enroll in ObamaCare and the taxpayer will pay their insurance cost
	#Obamacare is a #HUGE Success! Run on that record!	@BarackObama I rather pay penalty then enroll to expensive #obamacare sucks
Tweets at state-based marketplaces (eg, @NYStateofHealth, @MarylandConnect)	Thanks to @CoveredCA, my mom has insurance for the 1st time in 3 years &amp; can get new glasses for the first time in +10 years #ACA #thankyou	@NMHIX -Obamacare is a joke. Should trash what is left of it and leave the folks alone to figure their needs.
	@AccessHealthCT I am excited to share that we will be having an ACA recognition brunch this weekend here at CHC-Middletown #enrollCT	Concerning how I CANT get #ObamaCare in #Maryland NO customer service for password issues. BIG #FAIL for @MarylandConnect
Tweets at HealthCare.gov (@HealthCaregov)	@HealthCareGov I found a plan (or two) for less than $50... praise God, cause lord knows I'm broke phi broke. #ACA #ThankYou	@HealthCareGov @BarackObama How is Obamacare going to work if u can't even get the website or the enroll by phone to work? I want to sign up
	Best part of my week: When I took out my wallet for copay at the dr. and was reminded checkups now free. Thanks @HealthCareGov! #ACA	@HealthCareGov @WhiteHouse Obamacare is anti-choice and hurts people…

## Discussion

### Principal Findings

In our study conducted during the first open enrollment period of the health insurance marketplaces, we identified a significant correlation at the US state level between the ACA-related Twitter sentiment and the percent of eligible individuals who enrolled in a marketplace plan under the ACA. This correlation indicates the potential for Twitter to be a real-time monitoring strategy for future health insurance marketplace enrollment periods. Marketplaces could leverage systematic tracking of Twitter sentiment through commercially available software packages to more rapidly identify signals that indicate changes in marketplace enrollment, including emerging issues [23]. For example, a down-trending Twitter sentiment may indicate a problem in the marketplace, which could be further delineated by examining the content of negatively skewed tweets.

### Strengths and Limitations

Our study was limited by examination of a single month of Twitter and marketplace enrollment data, though March 2014 enrollment surpassed all prior months [21]. Further analyses are needed to assess if the correlation remains robust in the next open enrollment period and if content analyses could be used to address emerging issues in real time. Additionally, non-geocoded tweets that were excluded from analysis may have differed from those that were geocoded, though the absolute Twitter sentiment for each group was similar. Finally, further examination of states in which Twitter sentiment and marketplace enrollment were discordant are needed.

Of note, some studies using social media have recently come under scrutiny for manipulating people and violating their privacy [24,25]. Concern for such research was directed toward both the use of private data and the performance of an intervention without a clear informed consent. Neither of those properties apply to this study: we used public archival data and did not attempt to intervene on Twitter users’ lives. We believe studies like ours, which simply present statically grounded observations over public data, pose no risk to individuals and present no ethical concerns. Our study was approved by the University of Pennsylvania’s Institutional Review Board under “exempt” status.

### Conclusions

Twitter is a repository of free and accessible consumer-generated opinions [26]. Our study adds to a body of work indicating that Twitter is an emerging part of the health and health policy landscape [15,27-30]. The novel methodology used in our study linking Twitter sentiment to ACA implementation data may be an innovative way to inform how to improve the health care system in real time and may be applicable to other settings as health policy is implemented [15]. As the public debate over the ACA continues and federal and state marketplaces deal with the November 2014 open enrollment session, those managing the marketplaces and monitoring the ACA rollout may consider adding Twitter to their data and evaluation toolkit.

## Multimedia Appendix 1

Twitter handles for state-based health insurance marketplaces.

## Abbreviations

ACA: Affordable Care Act
NRC: National Research Council
