# Supplementary material for: Twitter Sentiment Predicts Affordable Care Act Marketplace Enrollment
Source: J Med Internet Res. 2015 Feb 23;17(2):e51. doi: 10.2196/jmir.3812 (PMC4376155; doi:10.2196/jmir.3812)
Supplement: Supplementary file 1 [file jmir_v17i2e51_app1.pdf]

Multimedia Appendix 1. Twitter Handles for State-Based Health Insurance Marketplaces.

State	Twitter Handle	# Tweets generated by account[a]	# Tweets at account handle[a]
California	@CoveredCA	534	8127
Colorado	@C4HCO	79	552
Connecticut	@AccessHealthCT	226	1415
District of Colombia	@DCHBX	3	21
Hawaii	@HIConnector	84	91
Idaho	@YourHealthIdaho	26	47
Kentucky	@kynectky	74	874
Maryland	@MarylandConnect	345	951
Massachusetts	@HealthConnector	83	285
Minnesota	@MNsure	13	1120
Nevada	@NVHealthLink	13	485
New Mexico	@NMHIX	99	287
New York	@NYStateofHealth	495	2000
Oregon	@CoverOregon	42	1471
Rhode Island	@HealthSourceRI	68	295
Vermont	@VTHealthConnect	45	242
Washington	@WAplanfinder	186	1080

[a]Tweets generated between March 1-31, 2014.
